# The interplay between mitochondria, the gut microbiome and metabolites and their therapeutic potential in primary mitochondrial disease

**DOI:** 10.3389/fphar.2024.1428242

**Published:** 2024-07-25

**Authors:** Kassandra A. Zachos, Jann Aldrin Gamboa, Aleena S. Dewji, Jocelyn Lee, Sonya Brijbassi, Ana C. Andreazza

**Affiliations:** ^1^ Department of Pharmacology and Toxicology, University of Toronto, Toronto, ON, Canada; ^2^ Mitochondrial Innovation Initiative, MITO2i, Toronto, ON, Canada; ^3^ Department of Psychiatry, University of Toronto, Toronto, ON, Canada

**Keywords:** mitochondria, mitochondrial disease, microbiome, mitochondria-microbiome crosstalk, diet

## Abstract

The various roles of the mitochondria and the microbiome in health and disease have been thoroughly investigated, though they are often examined independently and in the context of chronic disease. However, the mitochondria and microbiome are closely connected, namely, through their evolution, maternal inheritance patterns, overlapping role in many diseases and their importance in the maintenance of human health. The concept known as the “mitochondria-microbiome crosstalk” is the ongoing bidirectional crosstalk between these two entities and warrants further exploration and consideration, especially in the context of primary mitochondrial disease, where mitochondrial dysfunction can be detrimental for clinical manifestation of disease, and the role and composition of the microbiome is rarely investigated. A potential mechanism underlying this crosstalk is the role of metabolites from both the mitochondria and the microbiome. During digestion, gut microbes modulate compounds found in food, which can produce metabolites with various bioactive effects. Similarly, mitochondrial metabolites are produced from substrates that undergo biochemical processes during cellular respiration. This review aims to provide an overview of current literature examining the mitochondria-microbiome crosstalk, the role of commonly studied metabolites serve in signaling and mediating these biochemical pathways, and the impact diet has on both the mitochondria and the microbiome. As a final point, this review highlights the up-to-date implications of the mitochondria–microbiome crosstalk in mitochondrial disease and its potential as a therapeutic tool or target.

## 1 Introduction

It is widely understood that mitochondria are essential to normal body function and human health. Their role in the production and maintenance of energy is noteworthy, yet this organelle is imperative in many other mechanisms. Mitochondria are dynamic entities that act as signalling and integration hubs mediating biochemical pathways throughout the entire body (Whitaker et al., 2016). Due to their complexity, dysfunctional mitochondria are capable of provoking and contributing to profound cellular changes that can cause complications at the biochemical level (Aguilar-López et al., 2020). Causative factors of mitochondrial dysfunction encompass a wide array of possibilities: 1) damaged mitochondrial membranes, 2) the number of functional mitochondria, 3) an impairment in provision of substrates required for energy production or 4) genetic mutations that modulate the complexes along the electron transport chain. Since mitochondrial function is influenced by both the nuclear and mitochondrial genome, the precise mechanism is highly dependent on the individual, their environment, and their genetic makeup (Garagnani et al., 2014; Nicolson et al., 2018). Mitochondrial dysfunction may have systemic effects, however its damage is most prominent in organs with a high energy demand, such as the brain, heart, and muscles. Thus, it is plausible to assert that mitochondrial health plays a central role in brain health, aging, longevity, and immune response (Nicolson et al., 2018; Chen and Vitetta, 2019).

Growing evidence supports the pivotal role of the microbiome in maintaining human health and facilitating the development of chronic conditions, such as neurovegetative diseases (Forsythe et al., 2010; Moos et al., 2018). As exemplified in the Human Microbiome Project, the diversity of microbes within and amongst even the healthiest of individuals is remarkable (Human Microbiome Project Consortium, 2012). The link between the microbiome and human disease often stems from its diversity or lack thereof (Anhê et al., 2020). Many factors, including, but not limited to, our diet, environment, lifestyle, medications, and genetics, contribute to the development and maturation of microbes colonizing our gut. Evidence suggests that specific genes within our nuclear genome, particularly those associated with metabolism and immune function, contribute to the determination of bacterial taxa in the gut (Foster and McVey Neufeld, 2013; Garagnani et al., 2014). Improved understanding of the microbiome has led scientists to consider humans as metaorganisms, leading to scientific advancements in new study areas, such as “the gut-brain axis,” and “nutritional mental health.” Communication between the microbiome and the nuclear genome has been established and many mechanisms in the areas above are still being explored (Kaplan et al., 2015; González Olmo et al., 2021).

**FIGURE 1:**
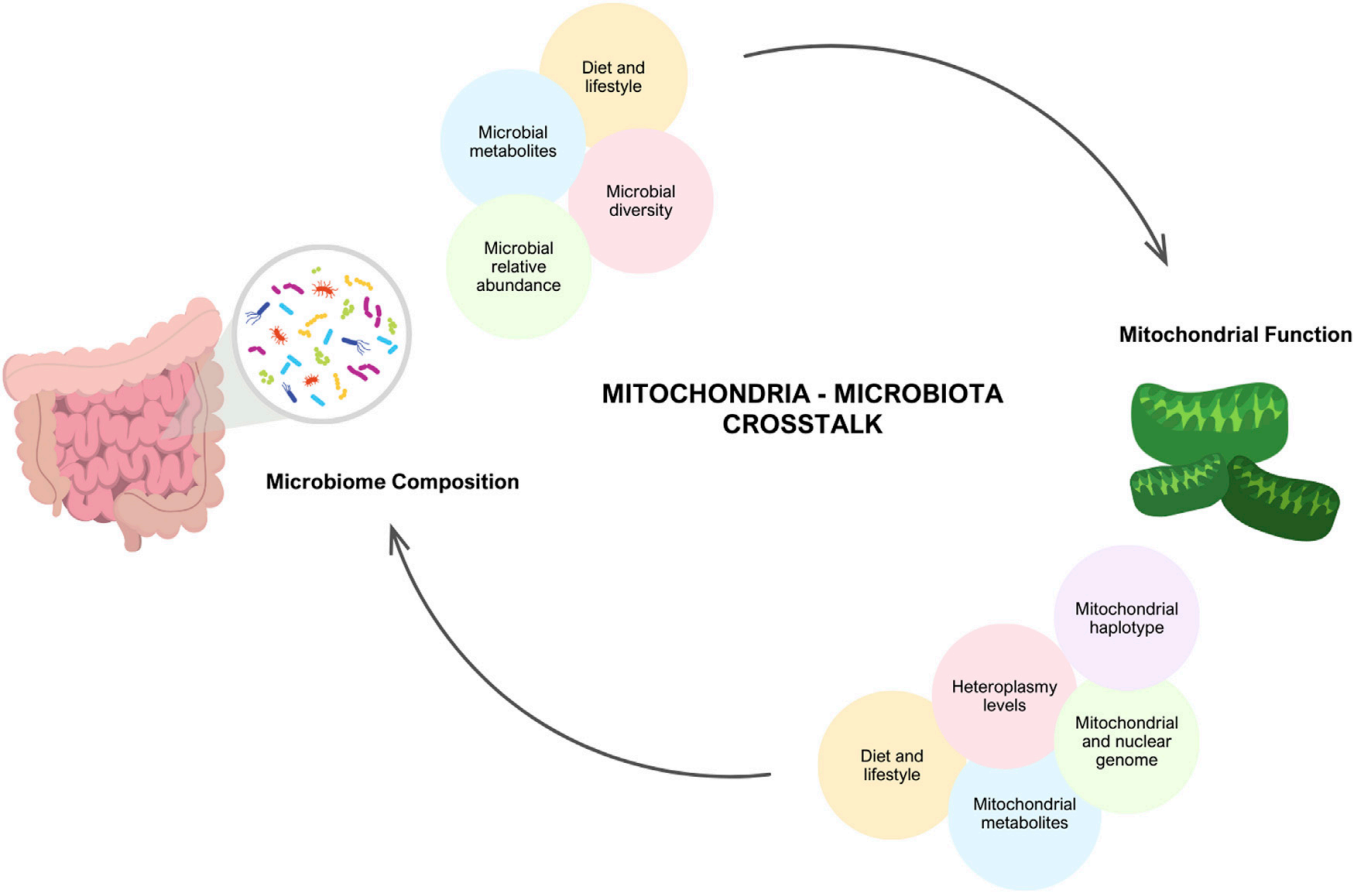
Interplay between Mitochondria and Gut Microbiome. There is evidence that there is communication between the mitochondria and the microbiome. Mitochondrial function is dependent on an individual’s genetics (mitochondrial and nuclear DNA mutations, heterpolasmy levels and the haplotype) and environmental factors (diet, lifestyle and stress). In turn, these factors have the potential to alter microbial diversity, relative abundance and microbial metabolites produced. Similarly, the microbiome composition and lifestyle factors (diet, exercise, medications) modulate mitochondrial function. Created with BioRender.com.

It is theorized that if both the mitochondrial genome and the microbiome interact with the nuclear genome, they are also likely to interact with each other, giving rise to the phenomenon known as mitochondria-microbiome interplay. (Figure 1) (Garagnani et al., 2014). From an evolutionary perspective, the mitochondrion is similar to microbes and is a known descendant of alpha-proteobacteria (Bajpat et al., 2018). Furthermore, both mitochondria and the microbiome are inherited maternally, mitochondria through the transmission of the mitochondrial genome, and the microbiome through prenatal colonization (Moos et al., 2018). This literature review aims to elucidate the intricate relationship between mitochondria and the microbiome by exploring their interplay, emphasizing the significance of their respective metabolites, and investigating the implications of diet, with a particular focus on mitochondrial disease.

## 2 Mitochondrial disease

Mitochondrial disease is an umbrella term for diseases and disorders in which mitochondrial dysfunction occurs; however, these conditions can be further classified into primary and secondary mitochondria diseases (Niyazov et al., 2016; Craven et al., 2017). Mitochondrial diseases that occur due to genetic mutations in the mitochondrial genome or nuclear genome, and directly code for mitochondrial function, are considered primary mitochondrial diseases (Schapira, 2006; Alston et al., 2017). Primary mitochondrial diseases are thought to affect one in 5,000 individuals, although the incidence is expected to be higher due to challenges in proper diagnoses and limited reported data (Horan et al., 2012). They differ from other monogenic disorders in that they lack a consistent genotype-phenotype correlation (Alston et al., 2017). This inconsistency can be attributed to a combination of many factors: the inheritance pattern of the reported genetic mutation, the interactions between the mitochondrial and nuclear genome, an individual’s haplogroup (specific patterns of inherited polymorphisms that are commonly used to track ancestry), and an individual’s tissue-specific heteroplasmy (Niyazov et al., 2016; Alston et al., 2017). Heteroplasmy is especially pivotal in primary mitochondrial diseases, as there is a variable threshold at which the cell can tolerate defective mitochondrial DNA. It is when the mutation load exceeds this threshold, we see metabolic dysfunction and clinical symptoms associated with a disease state (Niyazov et al., 2016). Secondary mitochondrial diseases are more loosely defined and even harder to diagnose as they can arise from environmental triggers and mutations that do not directly encode mitochondrial function. In many cases, secondary mitochondrial diseases occur when mitochondrial dysfunction is a downstream consequence of the initiating disease processes (Niyazov et al., 2016; Murphy and Hartley, 2018).

## 3 The mitochondria and mitochondrial metabolites on the microbiome

Mitochondrial dysfunction has been reported to influence microbiome composition and thus, it is function. Yardeni et al. (2019) detailed that the gut microbiome compositions of C57BL/6J mouse models, each characterized by distinct mitochondrial mutations, exhibited correlations with their respective genotypes (Yardeni et al., 2019). Using a cross-fostering approach, the microbial profile of pups were shown to closely resemble that of the foster mother, reflecting environmental influence. However, as the pups matured, the microbial profile reverted toward the distinctive microbial signature associated with their mitochondrial genotype. A separate study found that mitochondrial dysfunction promotes gut dysbiosis in the context of IBD. Specifically, these authors found that transplantation of fecal matter from mice deficient in the mitochondrial protein methylation-controlled J (MCJ) protein to germ free mice resulted in the transferring of the increased enteric inflammation that is observed in the MCJ deficient mice (Peña-Cearra et al., 2023). Other experiments supported that increased mitochondrial reactive oxygen species (ROS) production was associated with reduced microbial diversity, which then improved when ROS production decreased, as highlighted in Table 1. The connection between the gut microbiome, the mitochondria and ROS production has been further explored in a separate review (please refer to (Ballard and Towarnicki, 2020) for details).

**TABLE 1:** Critical mitochondrial metabolites with various signaling functions and their potential implication on the microbiota composition and function.

Mitochondrial metabolites	Function	Effect on the microbiome
**Reactive Oxygen Species (ROS)**	- Role in signaling and cellular balance at lower levels - Cell damage and interference in cell signaling (Ballard and Towarnicki, 2020)	- High levels contributes to microbial dysbiosis and reduced bacterial diversity in the gut microbiota - Facilitate mobilization of resident stem cells to support epithelial integrity (Ballard and Towarnicki, 2020)
**Lactate**	- Molecule used for glycogenesis and gluconeogenesis in the liver (acts as an energy source) - Signaling molecule in the brain that is associated with long-term memory *in vivo* - Lactate transport as a result of exercise regulates muscle acidosis (Ballou et al., 2016; Lee et al., 2021)	- Can inhibit the growth of some pathogens such as *E. coli* O157 (McWilliam Leitch and Stewart, 2002) - Lactate-utilizing bacteria can promote the growth of butyrate and propionate-producing bacteria (Sheridan et al., 2022) - However, it has also been reported that lactate of host origin can support the growth of certain pathogens such as *Salmonella* and C. albicans, which has been found to supress the production of butyrate (Gillis et al., 2018) - Increased levels of D-lactate can exacerbate symptoms of chronic fatigue syndrome (CFS) in patients due to its direct or indirect neurotoxic effects - High concentrations of lactate has been associated with some colonic disorders such as colitis and bowel syndromes (Taylor et al., 2022) - High concentrations of fecal lactic acid were related to lower risks of dementia (inverse association with the presence of dementia which indicates that lactic acid can be protective against dementia) (Wang et al., 2014)
**Succinate**	- A substrate in the citric acid cycle that plays a role in oxidative metabolism by interacting with mitochondrial electron transport chain to produce ATP (Matheoud et al., 2019) - Signaling molecule in thermogenesis (Matheoud et al., 2019) - Plays a role in maintaining gut homeostasis as it is produced by the microbiota and the host (Ferreyra et al., 2014) - Produced during bacterial fermentation of dietary fibers	- Plays a role in intestinal and extra-intestinal diseases that are associated with dysbiosis of the microbiota - Host and microbiome-derived - There is a link between dysbiosis, succinate accumulation and inflammation; Increased succinate can stimulate pro-inflammatory states by polarizing macrophages and increase intestinal inflammation (Wei et al., 2023) - Succinate can exacerbate some diarrheal diseases - Accumulation is associated with Inflammatory Bowel Disease (IBD) (Serena et al., 2018; Monfort-Ferré et al., 2022) - High levels of circulating succinate is associated with obesity, possibly by altering the gut microbiome composition. (Ferreyra et al., 2014) - Can be targeted for the treatment of obesity (Ferreyra et al., 2014); succinate can improve glucose utilization in the context of obesity (Canfora et al., 2019) - Succinate also indirectly promotes colonization resistance from other invading pathogens but some have reported succinate’s ability promote the growth of a variety of gut microbiota, both commensal and pathogenic such as *Salmonella* (Yan et al., 2022)
**Cytochrome C**	- Released into the cytosol from mitochondria and triggers apoptosis by activating caspase 3 (a protease that enables cell death) - Plays a key role in ATP synthesis by participating in electron transport (Martínez-Reyes and Chandel, 2020)	- Altered function of mitochondria of mucosal cells is associated with intestinal diseases such as colorectal cancer or inflammatory bowel disease - IBD patients demonstrate decreased activity of electron chain transport and thus decreased ATP levels - Mitochondrial dysfunction in the gut is associated with intestinal inflammation, bacterial invasion, and epithelial cell apoptosis as a result of a compromised epithelial barrier (Novak and Mollen, 2015)
**Citrate**	- Synthesized in astrocytes with high concentrations found in cerebrospinal fluid - Acts as a substrate in the TCA cycle which works to produce ATP (Wu et al., 2020)	- Changes to the microbiome occur when ferric citrate is prescribed to patients with chronic kidney disease (CKD) - Experimental CKD rats treated with ferric citrate showed increased bacterial diversity at levels comparable to normal control rats (specifically, tryptophanase-possessing families in the gut increased) - Administration of ferric citrate prevented further inflammatory states in patients with CKD by increasing gut microbial diversity (Xu et al., 2021a) - Ferric citrate can be used as a beneficial treatment in CKD patients to improve kidney and blood pressure - Citrate is able to trap and feed Fe III) to some Gram-negative bacteria, thus promoting their growth (Wu et al., 2020)
**Fumarate**	- Intermediate in the TCA cycle - Dimethyl fumarate (DMF), a fumarate derivative, is used as a treatment in patients with multiple sclerosis - DMF detoxifies mycotoxins (Araújo et al., 2011)	- Evidence suggests that DMF may play a role in normalizing the composition of the gut microbiome in an effort to prevent neuroinflammation however, further research is necessary - DMF decreases intestinal permeability and maintains the integrity of the gut epithelial barrier - DMF also increases the diversity and richness of microbiota - DMF was found to inhibit the growth of some pro-inflammatory bacteria and promote the abundance of commensal, albeit controversial, bacteria in patients with MS (Diebold et al., 2022) - DMF stimulated the growth of some commensal bacteria and attenuated gut inflammation (Fu et al., 2023) - In one study, DMF did not majorly affect the mycobiome composition and diversity (Yadav et al., 2022)

Other mitochondrial metabolites include those produced in the tricarboxylic acid cycle (TCA), which play a role in thermogenesis, tumorigenesis and immune modulation (Martínez-Reyes and Chandel, 2020). Some examples of these metabolites include succinate, fumarate, citrate, succinate, cytochrome c and lactate (Table 1). Some of these metabolites, like succinate and lactate have been more thoroughly examined in the literature, while for the others, data is more limited. Despite succinate’s critical role in cell signaling, an accumulation, often caused by the inactivation of succinate dehydrogenase (SDH), has been associated with a pro-inflammatory state (Martínez-Reyes and Chandel, 2020). For instance, individuals with Inflammatory Bowel Disease (IBD) were observed to have increased concentrations of succinate-producing bacteria (Connors et al., 2018) which correlated with increased inflammatory abrasions (Macias-Ceja et al., 2019). Strikingly, some IBD patients were found to have a diminished abundance of succinate-feeding bacteria (Morgan et al., 2012). Another research group found comparable results in the context of obesity, showing increased succinate levels (Serena et al., 2018). Succinate-induced stimulation of HIF-1α, a critical curator of gut health (Kumar et al., 2020; Pral et al., 2021), is one of the key regulators of the inflammatory state (Connors et al., 2018). In addition, increased succinate produced by *Bacteroides thetaiotaomicron* has also been found to be a catalyst of pathogenic bacterial colonization (Curtis et al., 2014; Ferreyra et al., 2014). Together, these results suggest that high inflammatory phenotypes induced by succinate, which can be evident in various mitochondrial diseases, contribute to microbial dysbiosis.

Lactate is a unique metabolite, that was considered a by-product of metabolism for many years. It is now better understood that lactate is a critical component in metabolic homeostasis, acts to signal and mediate biochemical pathways and is generated under in several different conditions, including the gut microbiome composition (Brooks, 2020). There are several lactate-producing bacteria, that typically ferment various monosaccharides and this lactate is commonly utilized by short chain fatty acids as an energy source. There are several well-established reviews and reports on the role of lactate in the gut microbiome and thus, will not be further outlined here (Wang et al., 2020; Louis et al., 2022; McGuinness et al., 2022). However, is critical to highlight lactate in this review as lactate is very tightly linked to mitochondrial function. Typically, elevated lactate is linked to a shift from aerobic respiration, due to mitochondrial dysfunction or damage, and this lactate signals that the organism is utilizing other methods, such as anaerobic respiration, to compensate for the depletion in energy production (Brooks, 2020). Elevated lactate is also a biomarker assessed in the diagnostic process for individuals with primary mitochondrial diseases and thus is worth highlighting and conducting further studies of lactate-producing bacteria in this population (Schapira, 2006).

Fumarate, a metabolite of the TCA cycle, has been highlighted for its role in immune modulation and cancer (Araújo et al., 2011; Diebold et al., 2022). However, in the context of the gut microbiome, it has been examined the most in disease such as psoriasis, multiple sclerosis, covid, and even some cardiovascular diseases, and mostly in the form of dimethyl fumarate (DMF) (Diebold et al., 2022). DMF has some controversial findings as in some cases it has been highlighted to attenuate growth of some commensal bacteria, ultimately contributing to gut inflammation. Alternatively, in other findings it inhibits the growth of pro-inflammatory bacteria (Yadav et al., 2022; Fu et al., 2023). Cytochrome c has limited findings in the context of microbiome studies, but it is known for its role in activating caspases, encouraging cell death and releasing ROS(Martínez-Reyes and Chandel, 2020). It has been linked to altered mitochondrial function in inflammatory bowel diseases, which are known to have microbial dysbiosis, but no further links have been well established (Novak and Mollen, 2015). Lastly, citrate, a key mediator of the TCA cycle and a rate-limiting metabolite known for its role in lipid synthesis (Martínez-Reyes and Chandel, 2020). In the context of the mitochondria and the microbiome, citrate has been examined in chronic kidney diseases, where supplementation of ferric citrate has improved inflammatory states and led to increased diversity of the gut microbiome. This is potentially due to its ability to chelate iron (Fe III), which feeds Gram-negative bacteria and increase richness (Wu et al., 2020).

In a separate study using mice strains with an identical nuclear genetic background, C57BL/6J (B6.B6), and varying mitochondrial genetic backgrounds (B6. NZB, B6NOD and B6. AKR) demonstrated that improved oxidative phosphorylation resulted in increased ATP levels and was protective against colitis (Bär et al., 2013). A separate study took the opposite approach and utilized knockout mice lacking the OCTN2 carnitine transporter, which was reported to cause spontaneous colitis and inflammation. This carnitine transporter is a critical transporter of long-chain fatty acids into the mitochondria to be metabolized via beta-oxidation (Shekhawat et al., 2007). Results from Ma et al. (2014), indicate that there are specific mitochondrial haplogroups associated with microbiome compositions polymorphisms in the ND5 and cytochrome b mitochondrial genes, as well as the D-loop region, exhibited associations with stool taxonomies such as Roseburia, Eubacterium and Deltaproteobacteria, respectively.

The microbiome of a mouse strain (C57BL/6J-mtFVB/NJ) carrying a mitochondrial mutation in the ATP-synthase 8 gene, was characterized and compared to other mice strains (Hirose et al., 2017). These mice had reduced beta diversity and altered microbiomes compared to the other models, specifically showing an increased relative abundance in Bacteroidales, Deferribacteraceae, Desulfovibrionaceae, and Helicobacteraceae. C57BL/6J-mtFVB/NJ mice underwent metabolic characterization, revealing that mitochondrial OXPHOS impairment was compensated by an upregulation in glycolysis. This suggests that mitochondrial impairments play a role in modulating the composition and function of the microbiome, as demonstrated by Hirose et al. (2017). Existing literature regarding the mitochondria’s role warrants further exploration. Studies may consider examining mitochondrial metabolites altered in primary mitochondrial diseases and assessing modulations on the microbiome function.

To our knowledge, one intervention study has been conducted in a population of adults with mitochondrial disease. This study was designed to address gastrointestinal dysmotility, an uncomfortable symptom in many mitochondrial diseases (Houghton et al., 2022). This study was a single-arm pilot trial where 24 patients completed a 12-week low-residue fiber diet. Overall, this diet was well tolerated, and differences were highlighted in improvements in stool consistency, reduced use of laxatives and improved overall GI discomfort. The gut microbiome was evaluated in these participants and in 10 non-disease controls, and while there were observed differences between the patients with mitochondrial disease and controls, there were no significant changes in the patients’ microbiomes over the course of the diet. Finally, there were no observed difference in participants transit time and stool frequency (Houghton et al., 2022).

## 4 Microbiome and microbial metabolites on the mitochondria in the context of disease

The body of literature examining the implications of the microbiome on mitochondrial function is growing rapidly, with supporting evidence that mitochondrial modifications are often mediated by bioactive metabolites produced by the microbes within the gut (Bajpat et al., 2018). For instance, short-chain fatty acids (SCFAs) and secondary bile acids have been reported to regulate redox balance, energy production, and activate AMP-activated protein kinases—a proposed connection linking SCFAs to mitochondrial biogenesis (Donohoe et al., 2011; Kim, 2018). SCFAs and mitochondrial health may also be linked through the modulation of synthesis pathways for immune messengers, such as TLR-4, IL-6, TNF-α (Luu and Visekruna, 2019; Dou et al., 2022). In addition, a specific SCFA called butyrate has been demonstrated to rescue the respiration of colonocytes in germ-free mice models as they undergo fatty acid oxidation (FAO) to produce acetyl coenzyme A (acetyl-CoA) and facilitating ATP production (Table 2) (Franco-Obregon and Gilbert, 2017; Mollica et al., 2017).

**TABLE 2:** Metabolites produced by the gut microbiota that have reported modulations on mitochondrial function.

Microbial metabolites	General function	Effect on the mitochondria
**SCFAs (Butyrate)**	- Butyrate is an energy source for colonocytes resulting from fermentation in the large intestine by gut microbiota - Butyrate is a fatty acid oxidized in the mitochondria - Increases oxidative phosphorylation - Protects from insulin resistance and fatty liver - SCFAs modulate lipid and glucose metabolism and display antidiabetic effects (Monfort-Ferré et al., 2022)	- Targets hepatic mitochondria to revert insulin resistance in diet-induced obese mice - Improves fatty acid oxidation - Improves mitochondrial cell energy metabolism - Indirectly combats obesity, fat accumulation and insulin resistance (Zhao et al., 2021; Monfort-Ferré et al., 2022)
**Phytoestrogens (isoflavones and flavonoids)**	- Shown to have estrogenic and antiestrogenic effects (Kossoff et al., 2009) - Flavonoids are plant secondary metabolites known for their benefits on human health: antioxidants, anticarcinogenic, antibacterial, anti-inflammatory, and antidiabetic (Kyriazis et al., 2022) - Consumption of flavonoids significantly decreases risk of cardiovascular diseases, breast cancer, and osteoporosis (Patterson and Sears, 2017)	- Flavonoids can modulate and ameliorate mitochondrial function to contribute to cytoprotection (Kossoff et al., 2009) - Interacts with potassium channels of the inner mitochondrial membrane to increase K+ ions in the mitochondria - Flavonoids regulate apoptosis, reduce ROS which are usually a byproduct of mitochondrial oxidation cycles - Flavonoids stimulate mitochondrial biogenesis - Overall decrease in mitochondrial dysfunction which can be applied to human diseases (Kim, 2018)
**Bile acids**	- Regulate gut microbiome and lead to microbial/bacterial dysbiosis when levels in the intestine are low (Rietjens et al., 2016) - Facilitate digestion and absorption of lipids in the small intestine as well as regulate cholesterol, energy, and triglyceride homeostasis (Stojanov and Kreft, 2020) - Bile acids have antimicrobial properties that impact gut microbes (Rietjens et al., 2016) - Activate the innate immune system to regulate the composition of the gut microbiome (Rasbach and Schnellmann, 2008)	- Increased bile acid concentration can lead to apoptosis or necrosis by damaging mitochondria - Bile acids can induce mitochondrial toxicity by making the membrane more permeable and eventually leading to cell death (Park et al., 2022)
**TMAO (trimethylamine N-oxide)**	- Potent pro-inflammatory factor associated with mortality in patients with chronic kidney disease - Alters cholesterol metabolism (increases deposition in artery walls) by promoting atherosclerosis (Vidali et al., 2015) - Plays a role in CVD and neurological disorders (Srivastava et al., 2012) - Implicated in the prognosis of patients with heart failure (Zhang et al., 2018)	- Increased levels of TMAO found in patients with mitochondrial dysfunction when supplemented with L-carnitine (Vanhauwaert et al., 2015) - TMAO impairs mitochondrial energy metabolism in the heart - Increased levels of TMAO impair pyruvate and fatty acid oxidation in cardiac mitochondria and this can further exacerbate cardiovascular events (Marriage et al., 2004) - TMAO inhibits fatty acid oxidation in cardiac mitochondria which in turn decreases the energy produced by cardiac cell (Voicescu et al., 2016; Zhang et al., 2018)
**Curcumin**	- Influences the microbiota-gut-brain axis by indirectly acting on the CNS - Antioxidant and anti-inflammatory properties aid in prevention and treatment of neurodegenerative diseases (Diebold et al., 2022)	- Curcumin can scavenge ROS, retains mitochondrial membrane potential, enhances mitochondrial biogenesis and fusion activity (Peters et al., 2022) - Can attenuate mitochondrial respiration and biogenesis (Hao et al., 2021) - Can be used to treat neurodegenerative diseases by protecting CNS cells from mitochondrial dysfunction (Peters et al., 2022)

Similar results were obtained (Hu et al., 2020) but using the combined effects of butyrate and acetate in the context of Islet cell dysfunction. Zhao et al. (2020) also investigated butyrate’s (sodium butyrate) effects on hepatic mitochondria in hopes of finding ways to restore glycemic control in individuals with diabetes. Their team discovered that sodium butyrate was able to enhance mitochondrial biogenesis and bioenergetics by increasing beta-oxidation, counteracting oxidative stress, and modulating the gene expression of several genes implicated in mitochondrial function (Zhao et al., 2020). A study developed a lymphoblastoid cell model of autism spectrum disorder, with a subset of these cells that have a mitochondrial dysfunction, as mitochondrial dysfunction has been highlighted in autism. Butyrate was then introduced at differing concentrations (0.1, 0.5, and 1 mM) for 24 h or 48 h. Interestingly, these authors found that butyrate enhances mitochondrial function as evidenced by increased ATP production in the cell lines that exhibits mitochondrial dysfunction but not in the cell lines that demonstrates normal functioning of the mitochondria. In addition, 1 mM of butyrate also lead to increased expression of genes involved in mitochondrial fission (PINK1, DRP1, and FIS1) (Rose et al., 2018).

Butyrate and urolithin A, a bioactive metabolite of ellagitannins, enhance skeletal muscle respiratory capacity and improve microbiome diversity (Franco-Obregon and Gilbert, 2017; Zhang et al., 2019). Notably, comparable results were also observed across other disease states (Bachem et al., 2019; Tang et al., 2020; Zhao et al., 2020; Hao et al., 2021; Li et al., 2023). The relative concentration of SCFAs in various disease states has also been studied based on the relative abundance of SCFA-producing bacteria. A particular research group found that some patients with Multiple Sclerosis have a diminished abundance of SCFAs-producing gut bacteria, such as Faecalibacterium prausnitzii and Roseburia intestinalis (Melbye et al., 2018). Researchers have observed this trend in other conditions as well, including IBD (Joossens et al., 2011; Kumari et al., 2013) and cerebral hypoperfusion (Su et al., 2023). However, the relative abundance of SCFA and SCFA-producing bacteria in patients with primary mitochondrial disorders is yet to be investigated.

Despite holding great promise, it is important to consider that high doses of butyrate may not be ideal, as demonstrated by Xu et al. (2021b). This study found that sodium butyrate decreased body temperature of C57BL/6 mice models by altering mitochondrial metabolism in the brain, including the TCA cycle and glycolysis, and resulted in transient mitochondrial swelling. Upon closer examination, they also discovered proton leakage via the opening of the mitochondrial permeability transition pore, which ultimately caused disintegration of the mitochondrial potential (Xu et al., 2021b). Interestingly, they found that the intervention did not alter the gene expression of their models. Rather, they suggested that the above changes occurred due to alterations in post-translational modification or mitochondrial enzymes (Xu et al., 2021b). Additionally, Peters et al. (2022) found that *in vitro* butyrate administration did not affect mitochondrial bioenergetics and instead, aggravated mitochondrial ROS production and dysfunction and in the context of sepsis. Clinical benefits of intravenous butyrate administration *in vivo* were not observed (Peters et al., 2022).

In contrast, other phenolic compounds, such as fisetin and quercetin, are converted by the microbiota to alkyl catechols and reported to induce apoptosis in senescent cells, which has been shown to increase the lifespan of mice (Shiels et al., 2019). Other studies have shown that these compounds also counteract oxidative stress and improve mitochondrial health (Karuppagounder et al., 2013; Alexandre et al., 2020; Dai et al., 2022). Curcumin, a well-known polyphenol with antioxidant and anti-inflammatory properties (Perrone et al., 2019), also becomes more biologically active after microbiome metabolism (Table 2) (Di Meo et al., 2019), suggesting that a diverse microbiome plays an important role in achieving maximum effects from such compounds. Importantly, studies demonstrated that curcumin is able to attenuate mitochondrial respiration and biogenesis, possibly via PGC1 pathway, or PPARγ and TFAM activation, among others (Eckert et al., 2013; Hamidie et al., 2015).

Phytoestrogens, which resemble human estrogens in their chemical structure and biological activity, have also been reported to affect mitochondrial function (Zaheer and Akhtar, 2017; Klinge, 2020). The isoflavones commonly found in soy products, enistin and daidzin, are phytoestrogens that are metabolized by bacterial β-glucosidases into bioactive compounds (genistein and daidzein, respectively) and have inconsistent ramifications on mitochondrial function (Kalaiselvan et al., 2010). More specifically, there is evidence of genistein increasing ATP concentrations, restoring complex activity, and maintaining membrane integrity (Rasbach and Schnellmann, 2008; de Oliveira, 2016). These results may be attributed to the compound’s ability to increase the tricarboxylic acid (TCA) cycle turnover and restore redox balance in the mitochondria (Rasbach and Schnellmann, 2008). Other research groups have also investigated the potential applications of genistein in various disease states and found overall positive results (Farruggio et al., 2019; Li et al., 2022). Notably, Li et al. (2022) found that genistein increases Mfn2 gene expression, a regulatory protein of various pathways (e.g., Ras/MAPK, PERK) that promotes mitochondrial health (Li et al., 2022). While genistein’s role in modulating mitochondrial function is more commonly investigated, there is also evidence of daidzein increasing expression of representative OXPHOS genes, such as COX1, CYTB and ATP5 (Rietjens et al., 2016; Cady et al., 2020). Yoshino et al. (2015**)** determined that daidzein was able to regulate mitochondrial biogenesis through a sirtuin-1 (SIRT-1)-associated pathway whereby it directly activated the mitochondrial transcription factor A (TFAM) promotor (Yoshino et al., 2015; Kicinska and Jarmuszkiewicz, 2020). Isoflavones’ role as an antioxidant is reported to act through different mechanisms. In some studies, daidzein and genistein function as ROS scavengers themselves, however in others, they mediate an increase in the production of other antioxidants such as glutathione (Lee et al., 2006; Rasbach and Schnellmann, 2008; Qian et al., 2012; Kładna et al., 2016; Voicescu et al., 2016).

Negative implications of isoflavones include the inhibition of mitochondrial function through its interaction with complex III, although this has only been examined at the cellular level, as opposed to the whole-body level (Salvi et al., 2002). Genistein was also shown to induce mitochondrial swelling, a loss of the mitochondrial membrane potential, and trigger the release of accumulated calcium (Salvi et al., 2002). Moreover, this compound triggered mitochondrial dysfunction through the upregulation cytochrome C and ROS production (Franco-Obregon and Gilbert, 2017; Lee et al., 2019; Chan et al., 2022). These effects are desirable when targeting malignant cells as they could potentially lead to apoptosis. However, these effects remain harmful to healthy cells and consequently, individuals.

Hydrogen sulfide (H2S) is a by-product of amino acid digestion and negatively impacts mitochondrial function by inhibiting cytochrome oxidase, a complex in the electron transport chain (ETC.) (Leschelle et al., 2005; Franco-Obregon and Gilbert, 2017). However, this is only reported to occur at high concentrations, since low levels of H2S can be reduced to form sulfide and used as a substrate in metabolism (Goubern et al., 2007; Saint-Georges-Chaumet and Edeas, 2015). Bile acids also have varying effects on mitochondrial function and at high levels, can contribute to apoptosis and necrosis through the nucleotide oligomerization domain (NOD)-like receptor protein-3 (NLRP3) inflammasome complex and membrane permeability (Table 2) (Palmeira and Rolo, 2004; Donohoe et al., 2011). Finally, trimethyl N-oxide (TMAO) is a potent pro-inflammatory metabolite typically associated with the consumption of red meat and has demonstrated the ability to impair mitochondrial function through pyruvate and fatty acid oxidation (Table 2) (Velasquez et al., 2016; Vallance et al., 2018; Zhang et al., 2021). In addition to impacting the mitochondria directly, various metabolites produced by pathogenic bacteria have been shown to have significant effects. One notable study by Matheoud et al. (2019) revealed that lipopolysaccharides (LPS) from Gram-negative bacteria can be taken up by antigen-presenting cells, triggering an autoimmune reaction against crucial neurons in the brain. The researchers utilized Pink1^−/−^ mice for their experiments, building on (Matheoud et al., 2016) previous findings that the protein Parkin prevents mitochondrial antigen presentation. The study demonstrated that CD8^+^ T cells, responsible for the autoimmune response, specifically target mitochondrial antigens expressed by dopaminergic neurons, leading to the induction of parkinsonism (Matheoud et al., 2019). Subsequent experiments confirmed this association, as the administration of L-DOPA reversed the condition (Matheoud et al., 2019). Interestingly, another research group discovered that sodium butyrate administration can alleviate LPS-induced pathologies by modulating T-cell activation, as reported by Dou et al. (2022). Moreover, a separate study (Li et al., 2022)found that butyric acid, a derivative of sodium butyrate, may promote mitochondrial function, potentially by influencing macrophage polarization.

It is clear there is an abundance of literature on microbial metabolites and mitochondrial function. A previous review has summarized the clinical applications of various flavonoids in the context of several diseases where mitochondriopathy is implicated (Koklesova et al., 2021). However, it is limited in its application towards primary mitochondrial diseases, in which the mitochondrial function is greatly impaired and may result in altered effects of such compounds. To our knowledge, these metabolites have not been investigated in interventional studies to improve mitochondrial functions in individuals with mitochondrial disorders. One research group has found elevation in plasma TMAO in patients with mitochondrial disorders treated with oral l-carnitine (Vallance et al., 2018). However, carnitine’s and TMAO’s efficacy in treating mitochondrial dysfunction has not been systematically evaluated. Interestingly, a separate research group investigated blood levels of TMA and TMAO, as well as mitochondrial DNA copy number in persons with healthy and unhealthy western diet (Bordoni et al., 2022). These authors found that there are no significant differences in the levels of TMAO between these two groups, or mitochondrial DNA copy number. Moreover, they found no significant correlation between TMAO and mitochondrial DNA copy number. TMAO along with other enteric microbiome-derived metabolites’ potential in ameliorating mitochondrial dysfunction has not been deeply explored clinically, which should be the focus of future intervention trials.

### 4.1 Future directions: strengths, limitations and potential therapeutic interventions

All literature highlighted and discussed in this review highlights some of the research that has been conducted examining the relationship between mitochondria and the gut microbiome, but it is clear that further research is required before we can utilize this relationship as a therapeutic intervention in primary mitochondrial diseases. In fact, conducting research to assess and improve our understanding on the interplay between the mitochondria and the microbiota is not anticipated to be an easy feat. These two entities are highly complex and challenging to understand and utilize as therapeutic interventions when assessed individually. The mitochondrion has a double-membraned structure, and various heteroplasmy levels in different tissues, which poses challenges in therapeutic design and effectiveness (Tyynismaa and Suomalainen, 2009). The microbiota is highly variable, and the gut microbiota is only one of several sources in the body where bacteria resides (Flint, 2020). Challenges that can be anticipated in future research studies include designing studies that will help elicit causality. Participant enrolment and controlling interventions are only two of several anticipated challenges. Animal studies are a useful tool when assessing causality, however generalizing any findings should be done cautiously (Saccone et al., 1991; Tyynismaa and Suomalainen, 2009). Lastly, off-target effects and systemic consequences are potential therapeutic adverse effects that need to be considered, especially since the mitochondria are found throughout the entire body and both the mitochondria and microbiota have effects on other systems, through the gut-brain axis, immune system, and metabolism, to name a few (Flint, 2020).

Alternatively, improving our understanding on the mitochondria-microbiome crosstalk has the potential for profound effects on personalized therapeutics, and overall efficacy of individualized treatments we design. Firstly, utilizing mitochondrial heteroplasmy, haplogroups, and mitochondrial DNA in the assessment of gut microbiota profiles, may demonstrate trends that we can utilize in our understanding on the development, function and metabolites produced from the microbiome. The pursuit of microbiome studies at the single-cell level, using tools such as microfluidics, fluorescence-activated cell sorting, and laser capture microdissection, can provide detailed insight on the interactions between the mitochondria and specific gut microbes. The utilization of Artificial Intelligence (AI) can also help sort and understand connections with all of these trends we have highlighted in the field up until this point. Lastly, in the context of mitochondrial disease, therapeutics for these individuals are highly limited. The introduction of fecal microbial transplants has the potential to improve the gastrointestinal discomfort, mood related consequences and immune function of these individuals. Understanding the gut microbiota can potentially highlight trends in specific mitochondrial diseases and improve patients stratification in these complex diseases.

## 5 Conclusion

This review underscores the intricate and dynamic relationship between mitochondrial dysfunction and the gut microbiome, highlighting the potential role of mitochondria-microbiome crosstalk in the pathophysiology of mitochondrial diseases. The bidirectional interactions between mitochondrial metabolites and microbial byproducts not only influence systemic metabolic pathways but also hold significant implications for disease manifestation and progression. Given the emerging evidence, targeting this crosstalk presents a promising therapeutic avenue. Future research should aim to deepen our understanding of these interactions through more detailed mechanistic studies, which could pave the way for novel dietary and microbial-based interventions. Such strategies may offer significant benefits, improving clinical outcomes for individuals suffering from mitochondrial disorders. Embracing a holistic view of these interactions, integrating genomic, proteomic, and metabolomic data, will be essential for developing personalized medicine approaches that can effectively address the complexities of mitochondrial diseases.
